# The Senses of Birth intervention to decrease cesarean and prematurity rates in Brazil

**DOI:** 10.1002/ijgo.12765

**Published:** 2019-02-13

**Authors:** Sônia Lansky, Bernardo J. Oliveira, Eliane R.M. Peixoto, Kleyde V. Souza, Luísa M.M. Fernandes, Amélia A.L. Friche

**Affiliations:** 1 Department of Health, Belo Horizonte City Hall, Belo Horizonte, Brazil; 2 School of Education, Universidade Federal de Minas Gerais, Belo Horizonte, Brazil; 3 School of Medicine, Universidade Federal de Minas Gerais, Belo Horizonte, Brazil; 4 School of Nursing, Universidade Federal de Minas Gerais, Belo Horizonte, Brazil; 5 School of Public Health, State University of New York at Albany, Albany, NY, USA

**Keywords:** Cesarean, Child health, Childbirth, Evidence-based medicine, Health education, Maternal health

## Abstract

**Objective:**

To increase knowledge and promote cultural change toward valuing normal birth, and to lower rates of cesarean and unnecessary interventions during childbirth in Brazil via the Senses of Birth (SoB) exhibition.

**Methods:**

The SoB intervention targeted 22 621 participants in three Brazilian cities in 2015. The effects of the exhibition in knowledge, perceptions, and preferences regarding childbirth were analyzed in a multi-method study. Pre- and post-exhibition survey responses of 17 501 (77.0%) visitors, 1947 (8.6%) non-pregnant women, and all pregnant women (n=1287) were collected at the exhibition. A follow-up survey was completed by 555 (43.0%) postpartum women who had participated at SoB while pregnant. Univariate analyses were used to compare before and after changes.

**Results:**

There was a significant increase in knowledge about normal birth, varying from 10.0% to 25.0% among general visitors (*P*<0.001) and 27.3% to 42.0% among pregnant women (*P*<0.001). Perceptions and preferences for normal birth also changed, reaching 83.0% of general visitors and 87.4% of pregnant women.

**Conclusion:**

SoB was found to effectively improve knowledge about and preference for normal birth. Scaling-up the intervention might contribute to cultural change toward valuing normal birth, and might decrease the rate of unnecessary cesarean and premature birth in Brazil.

## 1 INTRODUCTION

The rate of cesarean delivery in Brazil has been increasing for the past 40 years, reaching 57% in 2014.^1^ Cesarean rates are associated with increases in preterm birth rates and iatrogenic prematurity.^2^ Different interventions are necessary to address such a complex and cultural public health problem. Many historic, ethical, political, economic, and sociocultural relationships have had an impact, and have resulted in the institutionalization of childbirth, such that 98% of childbirth occurs in a hospital.^3,4^ Furthermore, commercialization and medicalization of life, power relations in science, medicine, and gender, and the social representation of cesarean delivery as safe, fast, convenient, clean, and a painless procedure have contributed to the rise in cesarean delivery.^3,4^

Different interventions are necessary to address this problem. The WHO has published best practice recommendations on childbirth care to improve rates of normal birth, and recently suggested that 15% should be the reference rate for cesarean delivery.^5,6^ The most recent UN agenda, Sustainable Development Goals,^7^ calls for increased accountability by challenging health systems to identify and fight the preventable maternal and neonatal morbidity and mortality associated with the inadequate access to services, or “too little, too late”.^8,9^ It also questions the opposite extreme reality, “too much, too soon,” which may be associated with overmedicalization of normal prenatal, intrapartum, and postnatal care.^8,9^

Public policies^10^ and social movement initiatives have attempted to reverse this situation, with campaigns and social mobilization for the humanization of childbirth. However, cultural change cannot rely solely on norms of procedures and information campaigns; it also requires educational actions that promote critical thinking.

To contribute to cultural change to value normal birth, and reduce unnecessary interventions during childbirth, the Senses of Birth exhibition was structured as a cultural intervention and health education action to promote the debate and diffusion of evidence-based practices in childbirth and birth care.^11^ The intervention was designed to support cultural transformation toward normalizing vaginal birth. To appraise the potential cultural transformation of this intervention, the aim of the present study was to analyze the effects SoB on the knowledge, perception, and preferences of visitors to the exhibition about normal birth and other aspects related to childbirth care.

## MATERIALS AND METHODS

The present cross-sectional quantitative multi-centered study analyzed survey data collected through the Senses of Birth project during the exhibition in three Brazilian cities (Belo Horizonte, Rio de Janeiro, and Niterói) from March 1 to August 31, 2015. The study was part of the research project named “Senses of Birth: effects of the interactive exhibition in the perception changes on labor and childbirth,” approved by the Federal University at Minas Gerais IRB (COEP/UFMG, 934.472). All participants provided informed consent through a signed consent form.

Senses of Birth is an interactive exhibit that combines different languages (digital art with theatrical techniques) and media (videos, photos, scenarios, and panels) to engage and excite the visitor and promote critical thinking. The free exhibition was open to the public and set-up in five Brazilian cities (Rio de Janeiro, Brasília, Belo Horizonte, Niterói, and Ceilândia), receiving 42 170 visitors from March 1, 2015, to December 31, 2017.

The exhibition consists of five sections and a 40-minute interactive circuit (see^12^ for a video presentation). It begins with the visitor being “impregnated” with a full term baby image projected in the visitor’s belly. A birth plan is then given to the new pregnant person (a child, an adolescent, adult or elder person, including both men and women) as a tool to inform and promote critical thinking about good practices and enabling them to plan their childbirth delivery. The “pregnant visitor” is then directed to the “convenience store”, where a vendor tries to sell labor and childbirth products, ironically disclosing childbirth business interests such as “Mama Beauty Spa” and a kit for planned cesarean delivery.

Next, the pregnant visitors attends a controversial discussion among characters on six screens representing the usually stereotyped opinions of doctors, doula, midwife, friends, and family that influence women during pregnancy. The pregnant visitor then goes through an emotional experience, extenuated by tactile and auditory gadgets simulating the fetus’s path through the birth canal. The experience starts in a warm and cozy uterus with a placenta and an umbilical cord, where the participant hears the mother’s heartbeat and a baby voice, and exits through a vaginal canal. The visitor is welcomed in the “conversation section”, a meeting point where informative panels, info-graphics, photos, and videos are available. The circuit is designed, and the staff/educators who conduct the cultural mediation at the exhibition are trained, to touch minds and hearts.

Data collection from the SoB intervention was developed in two phases and comprised four study populations. The first collection phase involved general visitors and pregnant women attending the exhibition; the second phase involved a follow-up survey of the pregnant women after childbirth.

The first study population comprised visitors who responded to the following two questions on touch-screen monitors at the exit of the exhibition: What was your opinion about normal birth before visiting SoB? What is your opinion of normal birth after visiting SoB? Each question had five options based on a Likert scale (terrible, bad, no opinion, good, excellent).

The second study population comprised visitors who answered a structured questionnaire before and after visiting the exhibition to measure changes in knowledge, perception, and preferences about normal vaginal birth. The group was selected by random sampling of 20% of the visiting population aged 18 years or older who agreed to participate; the sample size assumed a prevalence of 50% for the outcome (preference to vaginal birth), with a β level of 5%.

The third study population comprised all pregnant women who visited the intervention and answered a specific self-administered questionnaire after experiencing the SoB intervention. The fourth study population comprised women who had participated at the SoB intervention during pregnancy and answered an online self-administered follow-up questionnaire after childbirth, collecting information about the birth process, utilization of good practices, and outcomes.

The study variables were chosen on basis of the theory of planned behavior.^13,14^ The demographic information collected included sex, age, marital status, race (self-reported), family income (in multiplies of Brazilian minimum wage, US $225), education level, and health insurance type. Healthcare variables included self-reported knowledge about normal birth, doula support, midwife care, the right to a companion during childbirth, non-pharmacologic methods for pain relief, rates of cesarean in Brazil, WHO and Ministry of Health recommendations on childbirth, obstetric violence, and birth plan. Additional healthcare variables included perceptions and feelings associated with childbirth, including fear, suffering, safety, and risks; preferences in childbirth, including normal birth and cesarean; and perceived ability to have a normal birth. For postpartum women, healthcare variables included use of a birth plan, midwife care during childbirth, doula care during childbirth, type of hospital during childbirth, type of birth, and gestational age at birth.

SPSS version 20 (IBM, Armonk, NY, USA) was used for statistical analysis. Variables were described by number (percentage) and stratified by the different study populations. Touch-screen data on before and after opinions about normal birth were assessed by κ statistics. The Pearson *χ*^2^ test was used to compare outcomes before and after the SoB intervention; a *P* value of 0.05 was considered to be statistically significant.

## RESULTS

**FIGURE 1:**
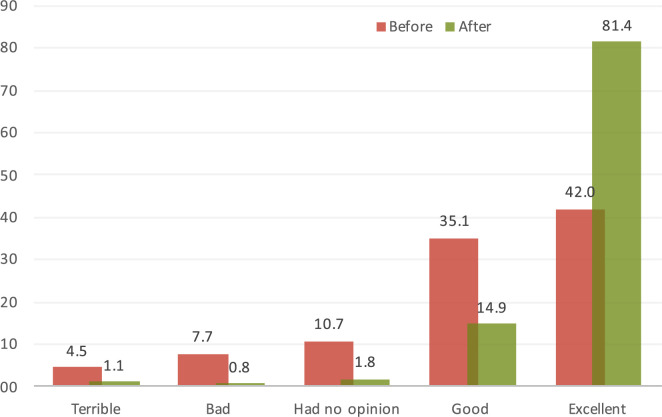
Visitors’ opinion of normal birth before and after visiting the Senses of Birth exhibition.

During the data-collection period, 22 621 visitors attended the SoB intervention and 17 501 (77.9%) provided touch-screen answers, which showed a substantial change in opinion about normal birth before and after visiting SoB. The percentage of people that reported their perception of normal birth as “terrible” (786; 4.5%), “bad” (1345; 7.7%), or “no opinion” (1877; 10.7%) decreased from 22.9% (n=4008) to 3.7% (n=641). The assessment of normal birth as “excellent” rose from 42.0% (n=7344) before to 81.4% (n=14 250) after the intervention (Fig. 1).

During the SoB, 1947 (8.6%) of the 17 501 visitors were interviewed: 1010 (51.9%) before and 937 (48.1%) after experiencing the intervention (Table 1). The general visitors (non-pregnant women) consisted of 428 men (22.0%) and 1518 women (78.0%). The majority were aged 20–34 years (1129; 58.4%), followed by 35 years or older (659; 34.1%) and 18–20 years (127; 6.6%). Most visitors were single (1107; 58.0%) and had a family income of 2–5 times the Brazilian minimum wage (636; 35.9%). The majority reported having health insurance coverage (1324; 69.2%) and having studied more than 12 years (1456; 75.5%); 379 (19.9%) had studied 8–12 years and 93 (4.8%) had less than 8 years of education. Roughly half (979; 50.9%) were self-reported black, 920 (47.8%) were white, and 25 (1.3%) were Asian, indigenous, or other race/ethnicity.

**TABLE 1:** Characteristics of the general population, pregnant and postpartum women that participated at Sense of Birth. Brazil, 2015.^a^

Characteristics	General population (n=1933)	Pregnant women (n=1287)	Post-partum women (n=555)
Sex			
Male	425 (22.0)		
Female	1507 (78.0)	1287 (100)	555 (100)
Age (years)			
≤19	127 (6.6)	68 (5.3)	32 (5.8)
20–34	1129 (58.4)	975 (76.5)	423 (76.9)
≥35	659 (34.1)	232 (18.2)	95 (17.3)
Skin color			
White	920 (47.8)	587 (45.9)	257 (46.6)
Black	979 (50.9)	665 (52.0)	284 (51.4)
Other	25 (1.3)	28 (2.2)	11 (2.0)
Marital status			
Single/separated/widowed	1107 (58.0)	265 (20.6)	86 (15.5)
Married or in union	802 (42.0)	1020 (79.4)	469 (84.5)
Income^b^			
<2 MW	417 (23.5)	282 (23.9)	102 (19.7)
2 to <5 MW	636 (35.9)	380 (32.2)	169 (32.6)
5 to <10 MW	444 (25.0)	293 (24.9)	135 (26.0)
≥10 MW	277 (15.6)	224 (19.0)	113 (21.8)
Schooling (years)			
<8	93 (4.8)	78 (6.2)	23 (4.2)
8–12	379 (19.7)	320 (25.4)	108 (19.6)
>12	1456 (75.5)	860 (68.4)	421 (76.3)
Health insurance			
Yes	1324 (69.2)	958 (74.6)	436 (78.8)
No	589 (30.8)	324 (25.2)	117 (21.2)

a Values are given as number (percentage). For some characteristics, the total varied owing to missing data.

b Multiples of minimum wage in 2015: $225.

Among 1287 pregnant women visitors, the majority were self-reported black (n=665; 52.0%), married (n=1020; 79.4%), and aged 20–34 years (n=975; 76.5%). Most had studied more than 12 years (n=860; 68.4%), and 959 (74.6%) were covered by private health insurance. A total of 517 (43.9%) women had a family income of more than five times the Brazilian minimum wage. The sample of postpartum women had characteristics similar to those of the pregnant women.

In terms of the visitors’ perceived knowledge about subjects related to childbirth, there were significant changes before and after the intervention among all three study groups (Table 2). The increase in perceived knowledge as good/very good among general visitors ranged from 10% (right to a companion, 586 [59.3%] to 640 [69.2%]) to almost 25% (Brazilian cesarean rate, 482 [48.6%] to 678 [73.1%]). Overall, before participating in the intervention, pregnant women self-reported greater knowledge about healthcare topics as compared with the general population. After participating in the intervention, the change in perceived knowledge was greater among pregnant women, ranging from 27.3% (knowledge of normal birth, 862 [68.0%] to 1212 [95.3%]) to 42.0% (knowledge about WHO recommendations for childbirth care, 525 [40%] to 1051 [82.5%]). Changes in knowledge reported by postpartum women were similar to those reported by pregnant women.

**TABLE 2:** Perceived knowledge Before and After the *Senses* of *Birth* Intervention, Brazil, 2015–17.

Variables	General Public			Pregnant Women			Post-partum women		
	Before (N=1000)^*^ n(%)	After (N=933)^*^ n(%)	*P*-value	Before (N=1287)^*^ n(%)	After (N=1287)^*^ n(%)	*P*-value	Before (N=555)^*^ n(%)	After (N=555)^*^ n(%)	*P*-value
Normal Birth									
None /Poor/ Fair	355 (36.2)	167 (17.9)	<0.001	405 (32.0)	60 (4.7)	<0.001	156 (28.3)	16 (2.9)	<0.001
Good /Very Good	625 (63.8)	764 (82.2)		862 (68.0)	1212 (95.3)		395 (71.7)	534 (97.1)	
Cesarean									
None /Poor/ Fair	469 (47.6)	319 (34.4)	<0.001	563 (45.0)	224 (17.9)	<0.001	217 (40.3)	63 (11.7)	<0.001
Good /Very Good	516 (52.4)	608 (65.6)		688 (55.0)	1030 (82.1)		321 (59.7)	477 (883)	
Risks of Normal Birth									
None /Poor/ Fair	477 (48.5)	289 (31.2)	<0.001	520 (40.9)	151 (11.9)	<0.001	193 (35.2)	47 (8.5)	<0.001
Good /Very Good	506 (51.5)	637 (68.8)		750 (59.1)	1121 (88.1)		355 (64.8)	504 (91.5)	
Risks of Cesarean									
None /Poor/ Fair	487 (49.5)	263 (28.5)	<0.001	547 (43.2)	169 (13.4)	<0.001	199 (36.4)	49 (9.0)	<0.001
Good /Very Good	496 (50.5)	659 (71.5)		718 (56.8)	1090 (86.6)		348 (63,6)	497 (91,0)	
Doul									
None /Poor/ Fair	679 (68.8)	448 (48.3)	<0.001	705 (54.9)	319 (24.3)	<0.001	270 (48.9)	103 (19.0)	<0.001
Good /Very Good	308 (31.2)	480 (51.7)		579 (45.1)	940 (74.7)		282 (51.1)	440 (81.0)	
Midwife									
None /Poor/ Fair	525 (53.0)	321 (34.7)	<0.001	615 (48.1)	243 (19.3)	<0.001	256 (46.4)	79 (14.6)	<0.001
Good /Very Good	465 (47.0)	604 (65.3)		664 (51.9)	1016 (80.7)		296 (53.6)	463 (85.6)	
Right to have companionship, from her choice, during the labor and childbirth									
None /Poor/ Fair	403 (40.7)	285 (30.8)	<0.001	434 (33.9)	182 (14.4)	<0.001	169 (30.7)	58 (10.6)	<0.001
Good /Very Good	586 (59.3)	640 (69.2)		845 (66.1)	1078 (85.6)		381 (69.3)	487 (89.4)	
Non-pharmacological birth pain relief methods									
None /Poor/ Fair	616 (62.0)	452 (48.8)	<0.001	737 (57.8)	278 (22.1)	<0.001	289 (52.5)	95 (17.5)	<0.001
Good /Very Good	377 (38.0)	474 (51.2)		539 (42.2)	982 (77.9)		262 (47.5)	449 (82.5)	
Humanized and evidence-base care during labor and childbirth									
None /Poor/ Fair	514 (51.8)	345 (37.3)	<0.001	668 (51.9)	250 (19.7)	<0.001	262 (47.5)	91 (16.6)	<0.001
Good /Very Good	479 (48.2)	579 (62.7)		616 (47.9)	1016 (80.3)		290 (52.5)	458 (83.4)	
Organizations that defend the humanized and evidence-base care / Humanization Movement									
None /Poor/ Fair	642 (64.7)	480 (51.6)	<0.001	839 (65.4)	336 (26.5)	<0.001	340 (61.5)	135 (24.6)	<0.001
Good /Very Good	351 (35.3)	451 (48.4)		444 (34.6)	930 (73.5)		213 (38.5)	414 (75.4)	
Brazil’s c-section rates									
None /Poor/ Fair	509 (51.4)	250 (26.9)	<0.001	625 (48.6)	142 (11.2)	<0.001	247 (44.6)	42 (7.7)	<0.001
Good /Very Good	482 (48.6)	678 (73.1)		661 (51.4)	1123 (88.8)		307 (55.4)	505 (92.3)	
Ministry of Health / WHO guidelines for labor and childbirth care									
None /Poor/ Fair	570 (57.6)	341 (36.7)	<0.001	747 (58.0)	221 (17.4)	<0.001	292 (53.2)	72 (13.1)	<0.001
Good /Very Good	420 (42.4)	589 (63.3)		525 (40.8)	1051 (82.6)		256 (46.7)	478 (86.9)	
Obstetric violence									
None /Poor/ Fair	641 (65.0)	419 (45.1)	<0.001	731 (57.7)	222 (17.5)	<0.001	286 (52.2)	71 (12.9)	<0.001
Good /Very Good	345 (35.0)	510 (54.9)		539 (42.3)	1050 (82.5)		262 (47.8)	480 (87.1)	
Birth Plan									
None /Poor/ Fair	695 (70.2)	484 (52.0)	<0.001	846 (66.6)	249 (19.7)	<0.001	334 (61.1)	94 (17.1)	<0.001
Good /Very Good	295 (29.8)	446 (48.0)		425 (33.4)	1017 (80.3)		213 (38.9)	455 (82.9)	

* Total varies according to missing information.

Concerning perceptions and feelings before and after the SoB intervention, respectively, the visitors indicated that vaginal birth was associated with happiness, personal fulfillment, safety, confidence, and strength, but negatively associated with fear, pain, suffering, and risk. The percentage of visitors who associated anxiety with normal birth decreased after participating at SoB (Table 3).

**TABLE 3:** Feelings and preferences associated with normal birth before and after the Senses of Birth Intervention.^a^

Feelings and preferences	General population			Pregnant Women			Post-partum women		
	Before (n=1000)	After (n=933)	*P* value	Before (n=1287)	After (n=1287)	*P* value	Before (n=555)	After (n=555)	*P* value
Feelings									
Happiness			0.003			<0.001			<0.001
Never/rarely/sometimes	293 (29.6)	219 (23.6)		467 (37.5)	150 (12.1)		167 (30.6)	57 (10.5)	
Frequently/always	696 (70.4)	710 (76.4)		777 (62.5)	1094 (87.9)		378 (69.4)	488 (89.5)	
Fear			0.005			<0.001			<0.001
Never/rarely/sometimes	692 (70.2)	702 (75.9)		813 (65.6)	1036 (83.5)		361 (66.5)	465 (85.6)	
Frequently/always	294 (29.8)	223 (24.1)		427 (34.4)	204 (16.5)		182 (33.5)	78 (14.4)	
Pain			<0.001			<0.001			<0.001
Never/rarely/sometimes	392 (40.1)	529 (57.3)		620 (48.8)	948 (76.1)		272 (50.1)	432 (79.6)	
Frequently/always	586 (59.9)	395 (42.7)		625 (50.2)	297 (23.9)		271 (49.9)	111 (20.4)	
Love			0.086			<0.001			<0.001
Never/rarely/sometimes	87 (8.9)	62 (6.7)		273 (22.0)	76 (6.1)		100 (18.5)	32 (5.9)	
Frequently/always	894 (91.1)	857 (93.3)		969 (78.0)	1166 (93.9)		442 (81.5)	510 (84.1)	
Suffering			0.006			<0.001			<0.001
Never/rarely/sometimes	765 (77.9)	763 (82.9)		861 (69.0)	1118 (89.6)		378 (69.2)	484 (88.6)	
Frequently/always	217 (22.1)	157 (17.1)		387 (31.0)	130 (10.4)		168 (30.8)	62 (11.4)	
Anxiety			0.012			<0.001			<0.001
Never/rarely/sometimes	431 (43.8)	457 (49.6)		725 (57.8)	959 (76.5)		312 (56.9)	426 (77.7)	
Frequently/always	552 (56.2)	464 (50.4)		529 (42.2)	295 (23.5)		236 (43.1)	122 (22.3)	
Safety			<0.001			<0.001			<0.001
Never/rarely/sometimes	352 (35.5)	240 (26.1)		518 (41.2)	167 (13.3)		208 (37.9)	68 (12.4)	
Frequently/always	639 (64.5)	679 (73.9)		740 (58.8)	1091 (86.7)		341 (62.1)	481 (87.6)	
Challenge			0.657			<0.001			<0.001
Never/rarely/sometimes	312 (31.5)	281 (30.5)		453 (36.1)	463 (36.9)		183 (33.4)	185 (33.8)	
Frequently/always	679 (68.5)	639 (69.5)		803 (63.9)	793 (63.1)		365 (66.6)	363 (66.2)	
Courage						<0.001			<0.001
Never/rarely/sometimes	162 (16.4)	132 (14.4)	0.226	308 (24.7)	308 (24.7)		135 (24.7)	142 (26.0)	
Frequently/always	828 (83.6)	787 (85.6)		941 (75.3)	941 (75.3)		412 (75.3)	405 (74.0)	
Risk									
Never/rarely/sometimes	817 (82.8)	812 (87.8)	0.002	1025 (82.4)	1098 (88.3)	<0.001	447 (82.5)	471 (86.9)	<0.001
Frequently/always	170 (17.2)	113 (12.2)		219 (17.6)	146 (11.7)		95 (17.5)	71 (13.1)	
Realization									
Never/rarely/sometimes	167 (16.9)	100 (10.8)	<0.001	337 (27.1)	96 (7.7)	<0.001	118 (21.8)	32 (5.9)	<0.001
Frequently/always	824 (83.1)	826 (89.2)		905 (72.9)	1146 (92.3)		424 (78.2)	510 (94.1)	
Confidence									
Never/rarely/sometimes	269 (27.3)	203 (21.9)	0.007	441 (35.4)	144 (11.5)	<0.001	170 (31.3)	55 (10.1)	<0.001
Frequently/always	718 (72.7)	724 (78.1)		806 (64.6)	1103 (88.5)		373 (68.7)	488 (89.9)	
Strength									
Never/rarely/sometimes	141 (14.3)	103 (11.1)	0.035	249 (19.9)	221 (17.7)	<0.001	85 (15.7)	99 (18.2)	<0.001
Frequently/always	844 (85.7)	825 (88.9)		1001 (80.1)	1029 (82.3)		458 (84.3)	444 (81.8)	
Preferences									
Normal birth preference									
No way/a little/maybe	203 (20.7)	154 (16.7)	0.026	136 (10.6)	–		40 (7.4)	–	
Probably/absolutely	696 (70.4)	710 (76.4)		1125 (87.4)	–		504 (92.6)	–	
Changed preference for normal birth									
No way/a little/maybe	–	313 (34.4)		–	475 (38.2)		–	204 (38.1)	
Probably/absolutely	–	586 (65.6)		–	770 (61.8)		–	331 (61.9)	
Cesarean preference									
No way/a little/maybe	832 (85.3)	823 (89.6)	0.006	1132 (93.4)	–		495 (95.4)	–	
Probably/absolutely	143 (14.7)	96 (10.4)		80 (6.6)	–		24 (4.6)	–	
Changed preference for cesarean									
No way/a little/maybe	–	353 (39.0)		–	645 (53.0)		–	302 (57.2)	
Probably/absolutely	–	551 (61.0)		–	571 (47.0)		–	226 (42.8)	
Consider herself able to have a normal birth									
No way/a little/maybe	248 (25.2)	157 (17.0)	<0.001	–	124 (9.8)		–	35 (6.4)	
Probably/absolutely	737 (74.8)	765 (83.0)		–	1137 (90.2)		–	516 (93.6)	

a Values are given as number (percentage). For some items, the total varied owing to missing data.

After the intervention, 586 (65.6%) general visitors reported that they had changed their perception of normal birth, and 551 (61.0%) had changed their perception of cesarean delivery. Before participating, 780 (79.3%) visitors reported that they probably or definitely preferred normal birth; this proportion changed to 770 (83.3%) after the intervention (P=0.027). Similarly, 14.7% (n=143) participants reported that they preferred cesarean delivery before the SoB, and this proportion decreased to 10.4% (n=96) after the intervention (P<0.05). Most visitors (737; 74.8%) reported that they could have a vaginal birth before visiting SoB; this proportion increased after the intervention (765; 83.0%). Among pregnant women, 61.8% (n=770) changed their opinion about normal birth; 87.4% (n=1125) declared a preference for vaginal birth after the intervention and 90.2% (n=1137) considered themselves able to have a normal birth after the intervention. The findings were similar among postpartum women (Table 3).

Among the 555 (43.1%) postpartum women who completed the follow-up survey, 354 (63.9%) reported that they delivered at a private hospital. In total, 255 (45.9%) had a cesarean delivery, 151 (59.7%) of them before labor. Notably, they reported use of a birth plan (306; 55.2%), midwife care during labor (234; 47.9%), and/or doula support (146; 26.9%). Overall, 330 (76.7%) had access to non-pharmacologic birth pain relief methods, 70 (13.2%) reported obstetric violence in childbirth, and 41 (7.4%) gave birth prematurely (Table 4).

**TABLE 4:** Childbirth characteristics among women who participated in the senses of birth intervention.^a^

Characteristic	Postpartum women (n=555)
Hospital type	
SUS-public	200 (36.1)
Private	354 (63.9)
Type of delivery	
Vaginal	300 (54.1)
Cesarean	255 (45.9)
Cesarean before labor	
Yes	151 (59.7)
No	102 (40.3)
Had doula support	
Yes	146 (26.9)
No	396 (73.1)
Had companion of choice in labor and childbirth	
Yes	453 (84.5)
No	83 (15.5)
Had a birth plan	
Yes	306 (55.2)
No	184 (33.2)
Didn’t know what birth plan is	64 (11.6)
Birth plan fulfilled	
Yes	221 (81.5)
No	50 (18.5)
Birth plan respected	
Yes	200 (64.9)
No	49 (15.9)
Had midwife care	
Yes	234 (47.9)
No	255 (52.1)
Non-pharmacologic pain relief
Yes	330 (76.7)
No	100 (23.3)
Had a previous pregnancy	
Yes	234 (47.9)
No	255 (52.1)
Gestational age at delivery, wk	
≤36	41 (7.4)
37–38	142 (25.8)
≥39	368 (66.8)
Reported obstetric violence	
Yes	70 (13.2)
No	460 (86.8)

Abbrevation: SUS, Sistema Único de Saúde (Unified Health System).

a Values are given as number (percentage). For some items, the total varied owing to missing data.

## DISCUSSION

The SoB educational intervention contributed to improvement in self-reported knowledge, as well as a change in perception and preferences about normal birth. In particular, knowledge about best practices recommended on the basis of scientific evidence and by the WHO and Brazil’s Ministry of Health increased significantly, especially among pregnant women. The degree to which participants had a positive perception about, and preference for, normal vaginal birth also increased after experiencing the intervention. Furthermore, even under the assumption that visitors to the SoB intervention might represent a particular group of individuals who favor normal birth, the percentage of individuals with a positive opinion (i.e., “good” and “excellent”) of vaginal birth increased from 77.1% to 96.3% after the intervention.

Knowledge about vaginal birth and the right to a companion during childbirth were the two indicators that changed the most among the general visitors. Studies have shown that a birth companion is the most valued right for women during childbirth.^15,16^ In Brazil in 2005, a law was passed guaranteeing every women’s right to have a companion of choice during all stages of labor in every public and private hospital or birth center without any additional costs to the patient^17^; nevertheless, this right is not guaranteed in all hospitals. In a national survey of women after childbirth, Diniz et al.^16^ found that 25% of women did not have a companion during their hospital stay, although only 6% reported that they did not want a companion.

Previous studies in Brazil have shown that fewer than 30% of women report a preference for delivery by cesarean at the beginning of pregnancy, but almost 90% of pregnancies result in cesarean even though only 10% have a medical indication.^2,18,19^ Studies also show that women in Brazil are not informed about best practices in childbirth during pregnancy.^20,21^ One study considers that women “accept” having a cesarean to avoid mistreatment and violent procedures during childbirth or obstetric violence.^20^ Domingues et al.^21^ reported that the most frequent reasons for avoiding a vaginal birth are fear of pain and mistreatment during childbirth and perceived inability to have a vaginal birth.

The rate of cesarean delivery is extremely high in Brazil, particularly in private hospitals (84.0%) and among high-income and highly educated women.^19^ Although the SoB postpartum population had similar characteristics, it had a lower rate of cesarean delivery and a lower rate of premature birth as compared with the national rates of 55.5% and 11.5%, respectively. Prematurity is a major concern, because it is the primary cause of neonatal death and also an important determinant of quality of life.^22^ Best practices are being implemented in public hospitals in Brazil, but there are challenges to change the private system to prevent unnecessary cesarean and iatrogenic prematurity.

Educational interventions are useful both for improving knowledge about childbirth best practices and for empowering women to make informed decisions. The SoB proved to be an effective initiative that contributed to improved knowledge about childbirth and was associated with use of evidenced-based practices and women’s right to a respectful birth. It has also promoted social mobilization through a social network with a website^23^ and 68 204 followers on Facebook, 4505 on Instagram, and 2900 on a YouTube channel.^24^

It is an inclusive, sensitive, and artistic intervention to engage society and disseminate accurate information to the population. Beyond the general public, it has specifically reached families with pregnant women, as well as adolescents, students and teachers of the public educational system, and health professionals at the primary healthcare and maternity level. These target populations are strategic to help change culture toward valuing normal birth as a tool to promote health and improve maternal and children indicators in Brazil. The main limitation of the study is that the visitors might not represent the general population. Neverthless, the study results showed a considerable change in knowledge, perception, and preferences of the visitors toward normal birth.

The SoB intervention contributed positively to improvement in knowledge about best practices in childbirth. It also influenced changes in feelings about and perceptions of normal birth. Scale-up of this initiative should be explored to support changes in culture toward valuing normal birth, and thereby promote maternal and child health in Brazil and decrease unnecessary cesarean procedures and premature birth.
